# Ectopic eruption of permanent first molars: a cross-sectional study in the mixed dentition

**DOI:** 10.1007/s00784-026-06905-9

**Published:** 2026-05-22

**Authors:** Marina Viana Scarpelli Aguiar, Lucas Gonçalves Santos, Cristiane Braga Barbosa Machado da Silva, Felipe Weidenbach Degrazia, Rodrigo Hermont Cançado, Leniana Santos Neves

**Affiliations:** 1 Department of Restorative Dentistry, area of Orthodontics, School of Dentistry, Federal University of Minas Gerais, Belo Horizonte, Brazil; 2 Department of Orthodontics, Federal University of Minas Gerais, Av. Pres. Antônio Carlos, 6627 - Pampulha, Belo Horizonte, MG 31270-901 Brazil

**Keywords:** Tooth Eruption, Ectopic, Molar, Tooth, Impacted

## Abstract

**Introduction:**

Ectopic eruption of permanent first molars can compromise arch development and lead to malocclusion if undetected. Identifying associated occlusal traits may enable early intervention and prevent complications.

**Objective:**

To determine the frequency and occlusal characteristics associated with ectopic eruption of permanent first molars in children.

**Materials and methods:**

A cross-sectional study analyzed 901 orthodontic files of children aged 5–9 years. Twenty-four patients with ectopic eruption of permanent first molars were included as the experimental group, while 23 age- and sex-matched children comprised the control group. Panoramic radiographs were used to assess molar angulation and digital dental models were used to assess arch length, arch perimeter, intercanine and intermolar distances. Data reliability was assessed using paired t-tests and Dahlberg’s formula. Statistical analysis included Shapiro-Wilk tests, independent t-tests or Mann-Whitney tests, Chi-square tests, and logistic regression to evaluate predictors of ectopic eruption.

**Results:**

Ectopic eruption was observed in 2.66% of children, predominantly in the maxilla. Children with ectopic eruption exhibited significantly shorter and narrower arches compared with control, with maxillary intermolar width emerging as a significant predictor (*p* < 0.05). Molar angulations and deciduous molar crown size were not significantly associated.

**Conclusion:**

Ectopic eruption of first permanent molars is associated with reduced arch dimensions, particularly maxillary intermolar width. Early recognition of these occlusal patterns may facilitate timely orthodontic management and prevent space loss and secondary crowding.

## Introduction

Ectopic eruption of permanent first molars is defined as an abnormal eruption pathway that results in impaction against the adjacent second primary molar. This condition may cause premature root resorption of the primary molar and compromise the alignment of the permanent dentition by reducing space for premolars [1]. Most cases are detected during the mixed dentition period, highlighting the importance of early diagnosis and timely interception to prevent malocclusions [2]. The scientific literature regarding the prevalence of this condition between sexes are scarce, suggesting no sexual dimorphism [3]. However, Chen et al. [4] and Zhang et al. [5] reported a higher prevalence in males, unilaterally or bilaterally, and is often associated with other dental anomalies, such as cleft lip and/or palate, supernumerary teeth, hypodontia, or infraocclusion of deciduous molars [3, 6, 7].

Reported prevalence varies from 0.75% to 4.3% with a clear predominance in the maxilla [8]. The etiology is multifactorial, involving both hereditary and local factors [2, 8, 9]. The hereditary factor accounts for approximately 19.8% of cases and has been related to insufficient maxillary growth [1, 5, 10, 11]. According to Sun et al. [12] and Zhang et al. [5], the angulation of the maxillary first permanent molar shows a close association with ectopic eruption, with a higher occurrence in Class III malocclusions presenting maxillary deficiency.

When diagnosis and treatment are performed at the appropriate time and in the correct way, unfavorable occlusal alterations can be prevented, consequently maintaining correct occlusion [13]. Radiographic examinations are recommended to identify abnormal eruptive positioning of permanent molars, with the aim of evaluating whether the tooth is impacted against the distobuccal root of the adjacent tooth. The ideal age for such evaluation is between five and seven years [2]. Moreover, clinical examination should assess whether the first permanent molar is erupting with an atypical inclination or failing to erupt within the usual timeframe [3]. Therefore, during pediatric dental visits, practitioners should carefully monitor the eruption of first permanent molars and routinely assess them through radiographs [14].

In addition to occlusal deviations, ectopic eruption of permanent first molars may lead to other pathological consequences, including damage to the adjacent second deciduous molar, such as pulpitis, premature root resorption, and mobility. In such situations, extraction of the deciduous tooth is often indicated [9]. Pain associated with the affected area may also occur, commonly related to resorption of the deciduous molar [15].

Ectopic eruption of permanent first molars can be classified as reversible, when spontaneous correction occurs, or irreversible, when intervention is required [4, 9, 16]. Rates of spontaneous corrections of ectopic eruption of permanent first molars in the maxilla range from 47% to 78% by the age of seven years [16]. However, the likelihood depends on predictive factors, such as the severity of atypical distal root resorption of the second primary molar, the degree of impaction, eruption angle, and bilateral occurrence [2]. Early intervention is therefore recommended in irreversible cases [4, 16, 17]. For mild cases, interproximal separation in the affected area may be proposed. However, when movement of more than 2 mm of the first permanent molar is required for disimpaction, distalization of the tooth or, in more severe cases, extraction of the adjacent second deciduous molar may be indicated [18, 19].

Therefore, the present study was designed with two main objectives: (1) to determine the frequency of ectopic eruption of permanent first molars in a pediatric orthodontic population, and (2) to analyze associated occlusal and arch characteristics, with the goal of identifying potential diagnostic indicators for early clinical intervention.

## Materials and methods

This cross-sectional study was approved by the institutional research ethics committee of Federal University of Minas Gerais (Process no. 89148225.5.0000.5149).

The sample of this study was acquired from the files of the Orthodontics Department at Federal University of Minas Gerais. From approximately 2,500 orthodontic records available between 2010 and 2024, a single research examiner selected a sample of 901 records corresponding to children aged 5 to 9 years, considering the eruption period of the first permanent molars. Records were screened to exclude duplicates from multiple visits of the same individual. From the selected sample, 24 patients were identified with ectopic eruption of permanent first molars, involving both maxillary and mandibular arches and unilateral or bilateral cases. This sample included all identified cases of the condition under investigation, thus characterizing a convenience sample. To investigate the associated occlusal characteristics, a group of 23 patients was selected, matched for age and sex, thereby comprising the control group. Model-based variables reduced due to unavailable digital models (*n* = 15 ectopic, *n* = 18 control for intermolar width).

Exclusion criteria were as follows: (1) Children should not present anomalies in tooth number, shape, or size; (2) Should not have experienced premature loss of deciduous teeth, nor present systemic anomalies or conditions; (3) Patients with prior orthodontic treatment.

All participants in both experimental and control groups were in the mixed dentition stage and possessed pre-treatment orthodontic records including panoramic radiographs. However, not all individuals had plaster models available, which reduced the sample size for the variables measured in models.

A statistical power analysis was conducted for the Student T test for independent samples using the variable maxillary intermolar distance, comparing the experimental group (*n* = 15, mean = 41.0, SD = 2.48) and the control group (*n* = 18, mean = 45.0, SD = 4.13). The test was two-tailed with a significance level set at 5% (α = 0.05). The purpose of this analysis was to ensure that the sample size and observed effect were sufficient to detect a true difference between groups, minimizing the risk of a type II error (false negative). The effect size, calculated using Cohen’s *d*, was 1.15, representing a large effect according to Cohen’s criteria [20]. With this effect size and the given sample sizes, even considering the limitations in comparisons based on plaster models, the estimated statistical power was 88.9%, indicating a high probability of detecting a real difference between groups, if presented.

**Fig. 1:**
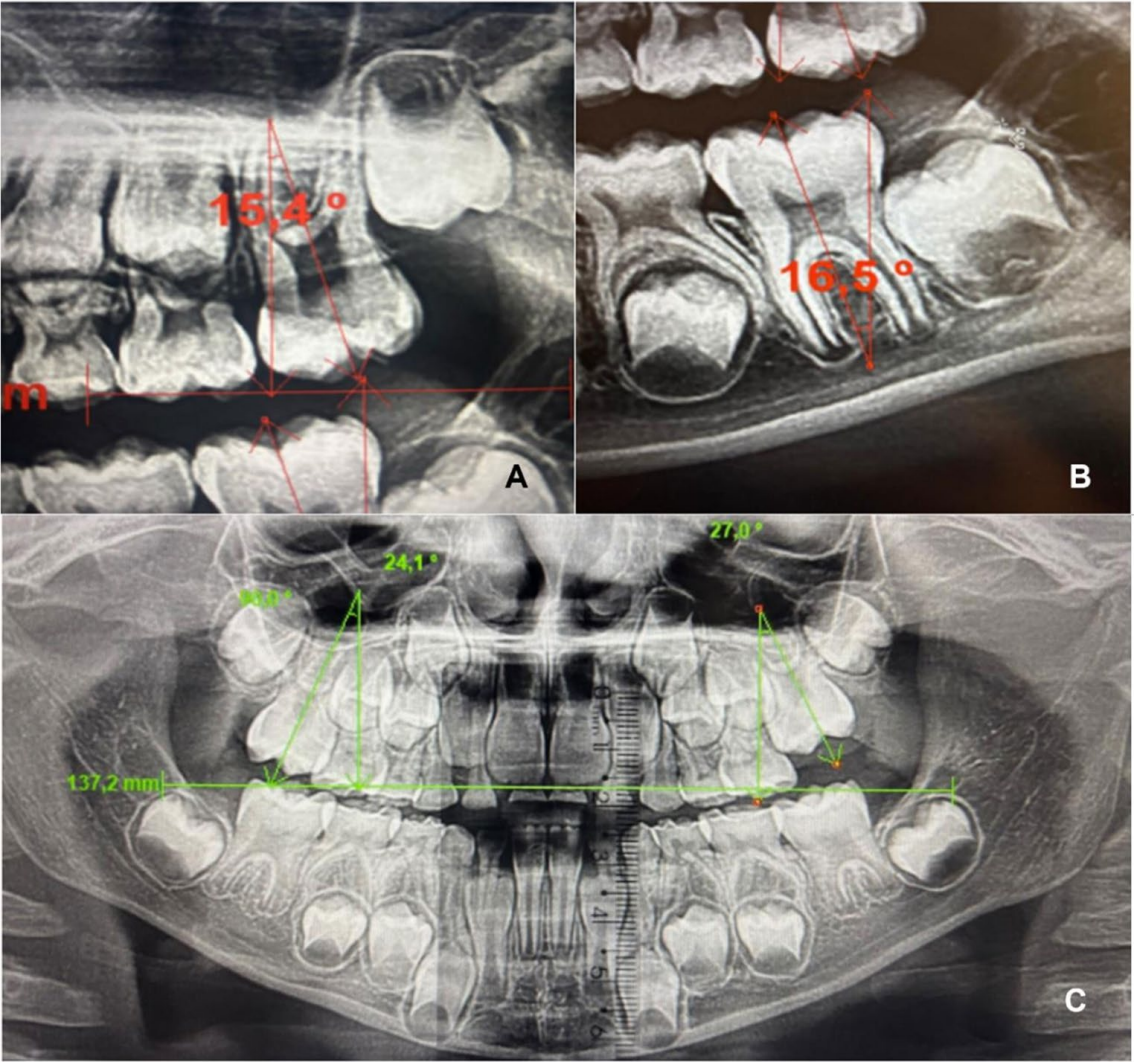
(**A**) Measurement of the long axis of the maxillary first molar; (**B**) Measurement of the long axis of the mandibular first molars; (**C**) Panoramic radiograph measurements of a study group patient performed with Radio Memory software (desktop version)

**Fig. 2:**
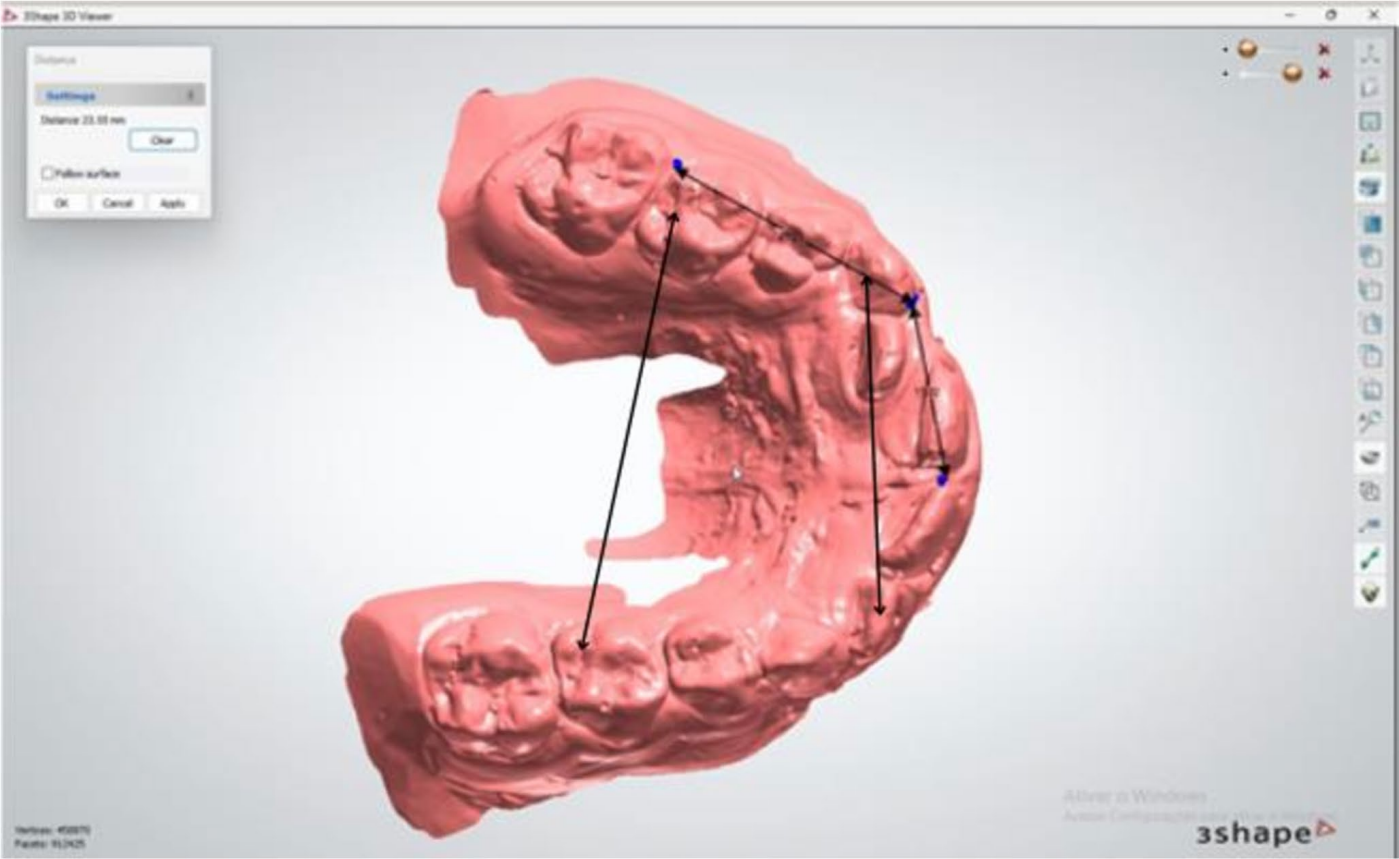
Model measurements of a study group patient performed with 3Shape 3D Viewer (v.1.4.1.1, Windows)

The following variables were assessed in both the experimental and control groups:

1. Radiographic assessment of first permanent molars angulation – measured on panoramic radiographs by a single examiner, assessing the long axis of the tooth in relation to a true vertical line perpendicular to the occlusal plane. Panoramic radiographs were obtained from a single standard orthodontic equipment available in the department during the period. To establish the long axis, the buccal groove was considered for maxillary molars and the mesiobuccal groove for mandibular molars, along with the furcation center for both maxillary and mandibular molars (Fig. 1). Prior to measurements, image resolution and magnification were standard using the software Radio Memory Studio version 3.0 Release 15.7, (Belo Horizonte, Brazil). Only radiographs with adequate contrast, minimal distortion, and no significant artifacts affecting measurements were included. Angulation was considered negative when the crown was positioned more distally relative to the root, and positive when the crown was positioned more mesially.
2. Digital dental models – dental models of the selected patients were scanned using the Primescan (Dentsply Sirona, USA, 2019). Measurements of arch perimeter, arch length, intercanine distance, and intermolar distance were performed by a single examiner (Fig. 2). Digital measurements were obtained with the 3Shape 3D Viewer software – version 1.4.1.1 for Windows (3Shape 3D Viewer, 3Shape A/S, Copenhagen, Denmark), as follows: mesiodistal widths of the second deciduous molars were measured between the mesial and distal contact points at the greatest convexity of the crown, as observed in the buccal view. Arch length was measured in the occlusal view, from a distal reference plane connecting the midpoints of the distal surfaces of the second deciduous molars to the anterior point located between the central incisors. Arch perimeter was measured in the occlusal view by summing the distances from the distal surface of the second deciduous molar to the mesial surface of the first deciduous molar, from the mesial surface of the first deciduous molar to the distal surface of the lateral incisor, and from the distal surface of the lateral incisor to the midline between the central incisors on both sides. Intercanine distance was measured in the occlusal view between the cusp tips of deciduous canines. Intermolar distance was measured in the occlusal view between the distal fossae of the second deciduous molars.

Molar angulation followed Zhang et al., 2024 [5] with long axis relative to reference planes on panoramic radiographs. Arch length, perimeter, intercanine, and intermolar widths followed standard digital model protocols [21].

Participant identification data were anonymized and coded to preserve confidentiality. However, since ectopic eruption is a visible condition, blinding of the study was not possible.

### Statistical analysis

*All statistical analyses were reviewed by a statistician experienced in clinical dental research*,* who confirmed the suitability of both parametric and non-parametric tests*,* as well as the use of univariate logistic regression*,* considering the sample size and the exploratory objectives of the study.*

The error of the study was assessed with 20% of the sample re-measured after a minimum interval of 15 days and compared with the initial measurements using the paired t-test (to evaluate systematic error) and Dahlberg’s formula (to evaluate random error) [22]. Descriptive statistics were employed with means, standard deviations, and frequency measures (percentages).

The Shapiro–Wilk normality test was applied to investigate data distribution. Additionally, the independent t-test was used for quantitative variables (age), *while the Chi-square test was applied for nominal variables (sex*,* mixed dentition stage*,* presence or absence of vertical - open bite and overbite – transverse – posterior crossbite malocclusions*,* and anterior mandibular crowding) to verify compatibility of these parameters between groups.* For comparison of study variables, the independent t-test or Mann-Whitney test were applied based on normality distribution.

Within the selected sample, two experimental subgroups were defined: one comprising maxillary right first molars affected by ectopic eruption, and another comprising maxillary left first molars affected by the same condition. The subgroups were created so that the angulations of only the affected teeth could be compared, since in the total sample there were cases of unilateral occurrence, sometimes on the right side, sometimes on the left, in addition to cases in the lower arch, where the upper arch did not present ectopic eruption. Thus, the angulations of affected tooth were compared in the control group using the independent t-test. Logistic regression analysis was performed to evaluate the influence of the main variables (predictor variables) on the occurrence of ectopic eruption of maxillary first permanent molars (outcome variable). The test was applied first to maxillary right first molars and subsequently to maxillary left first molars. Logistic regression was not applied to mandibular first molars due to the low frequency of occurrence of the condition in these teeth.

A significance level of 5% was adopted. Statistical analyses were performed using the Jamovi software, version 2.6.26 (Jamovi 2.6.26, 2025, Sydney, Australia).

## Results

None of the variables presented a systematic error, and the range of casual errors varied from 0,08 (55 tooth size) to 0,48 (maxillary perimeter).

Normal distribution was verified with Shapiro-Wilk test. Mandibular arch perimeter in the experimental group, mandibular intermolar distance in the experimental group, and maxillary intercanine distance in the control group presented statistical differences (*p* < 0.05), showing that these variables were not normally distributed. Table 1 presents the results of the compatibility tests performed between the groups, to assess whether they were comparable with respect to age, sex distribution, mixed dentition stage, vertical and transverse malocclusion, and anterior mandibular crowding. No statistically significant differences were found in either comparison, indicating that the groups were homogeneous regarding age, sex, mixed dentition stage, vertical malocclusion, and anterior mandibular crowding distribution (*p* > 0.05). However, transverse malocclusion was significantly more frequent in the ectopic eruption group (*p* = 0.010).

**Table 1:** Compatibility between experimental and control groups regarding age, sex, mixed dentition stage, transverse and vertical malocclusion and anterior mandibular crowding distribution with Independent t-test and chi-square test

Age – Independent t-test					
	Experimental Group		Control Group		
	Mean	S.D.	Mean	S.D.	**P**
Age	7.81	1.14	8.41	1.03	0.063^A^
Sex – Chi-Square Test					
	Experimental Group		Control Group		P
Male	16		15		0.916^B^
Female	8		8		
Mixed dentition stage – Chi-Square Test					
	Experimental Group		Control Group		P
1 st transitional period	8		5		0.063^B^
Intertransitional period	7		8		
2nd transitional period	0		5		
Transverse malocclusion – Chi-Square Test					
	Experimental Group		Control Group		P
Yes (present)	9		3		0.010^B^
No (absent)	6		15		
Vertical malocclusion – Chi-Square Test					
	Experimental Group		Control Group		P
Absent	8		9		0.942^B^
Open bite	5		7		
Deep bite	2		2		
Anterior mandibular crowding – Chi-Square Test					
	Experimental Group		Control Group		P
Yes (present)	7		10		0.898^B^
No (absent)	8		8		

^A^ Independent t-test

^B^ Chi-Square Test

From a total of 901 orthodontic files of children aged 5 to 9 years, 24 presented ectopic eruption of permanent molars, corresponding to a prevalence of 2.66%. 43.75% corresponded to the maxillary left first molar, 40.63% corresponded to the maxillary right first molars, 9.37% to the mandibular right first molar and 6.25% to the mandibular left first molar. Ectopic eruption of first permanent molars was more frequent in the maxilla (84.37%) compared with the mandible (15.63%). Among the individuals with this condition, 66.66% had involvement of only one tooth, while 33.33% presented involvement of two molars, always in the same arch.

Table 2 shows the results of the comparisons between experimental and control groups. Statistically significant differences were observed for the following parameters: maxillary arch length, maxillary intermolar distance, maxillary intercanine distance, mandibular arch length, mandibular intermolar distance, and mandibular intercanine distance. In all cases, the experimental group presented smaller measurements, indicating shorter and narrower arches in patients with ectopic eruption of first permanent molars.

**Table 2:** Comparison between experimental and control groups considering measurements performed on digital dental models

	Experimental Group (*n* = 24, digital models *n* = 15)		Control Group (*n* = 23, digital models *n* = 17)		*p*
	Média	DP	Média	DP	
16 Angulation	−14.0	9.81	−14.5	7.85	0.867^A^
26 Angulation	−13.5	11.9	−13.5	7.78	0.998^A^
36 Angulation	19.5	6.97	18.4	4.74	0.520^A^
46 Angulation	21.2	8.59	20.2	6.29	0.641^A^
Maxillary perimeter	77.7	3.99	79.5	3.83	0.187^A^
Maxillary length	26.6	2.72	28.7	2.58	0.029^A^*
Intermolar maxillary width	41.0	2.48	45.0	4.13	0.003^A^*
Intercanine maxillary width	32.0	2.54	33.8	2.89	0.017^B^*
55 Tooth size	9.78	0.779	9.46	0.479	0.195^A^
65 Tooth size	9.65	0.607	9.41	0.401	0.188^A^
Mandibular perimeter	71.9	3.94	73.9	3.44	0.186^B^
Mandibular length	24.3	2.62	26.6	2.44	0.015^A^*
Intermolar mandibular width	39.3	2.25	41.7	2.94	0.013^B^*
Intercanine mandibular width	25.6	3.10	27.8	2.55	0.040^A^*
75 Tooth size	10.4	0.742	10.6	0.603	0.526^A^
85 Tooth size	10.5	0.694	10.6	0.753	0.601^A^

^A^ Independent T-test

^B^ Mann-Whitney Test

* Statistically significant at *p* < 0.05

Table 3 presents the results of the comparisons of molar angulations. No statistically significant differences were found in the angulations of first permanent molars between the experimental and control groups, even when restricting the analysis to only the affected teeth within the experimental group. Therefore, the angulations of maxillary permanent molars with ectopic eruption were statistically similar to those of permanent molars without the condition.

**Table 3:** Comparison of the maxillary molar subgroups affected by ectopic eruption with the corresponding teeth in the control group

	Experimental Group- affected tooth		Control Group – Correspondent tooth (*n* = 23)		*p*
	Mean	S.D.	Mean	S.D.	
16 Angulation (*n* = 13 affected)	−9.30	9.16	−14.5	7.85	0.083
26 Angulation (*n* = 14 affected)	−8.49	12.0	−13.5	7.78	0.133

Independent T-test

Table 4 reports the results of the simple (binomial) logistic regression analyses, testing associations between the dependent variables and the independent variables. Simple logistic regression was applied to evaluate the association between each independent variable and the occurrence of ectopic eruption of maxillary first permanent molars. Among the arch perimeter and length variables and the maxillary intermolar and intercanine distances, only the maxillary intermolar distance significantly influenced the occurrence of ectopic eruption of maxillary first permanent molars (*p* = 0.0365). In this univariate logistic regression, maxillary intermolar distance emerged as the sole significant predictor with an odds ratio of 0.7481 (95% CI: 0.57–0.98), suggesting that for each 1 mm increase in intermolar distance, the odds of ectopic eruption decreased by approximately 25.2%.

**Table 4:** Results of the simple (binomial) logistic regression analysis

Variables	Coeficient	Standard error	Z	*p*	Odds ratio	IC 95%
Outcome: 16 impaction Predictor: 16 angulation						
Intercept	0.3481	0.6347	…	…	…	…
X1	0.0770	0.0457	1.6853	0.0919	1.0800	0.99 a 1.18
Outcome: 16 impaction Predictor: 55 size						
Intercept	−11.9985	7.2818	…	…	…	…
X1	1.1470	0.7434	1.5429	0.1229	3.1487	0.73 a 13.52
Outcome: 26 impaction Predictor: 26 angulation						
Intercept	0.1205	0.5311	…	…	…	…
X1	0.0558	0.0376	1.4860	0.1373	1.0574	0.98 a 1.14
Outcome: 26 impaction Predictor: 65 size						
Intercept	−13.3920	10.4906	…	…	…	…
X1	1.3543	1.0988	1.2326	0.2177	3.8741	0.45 a 33.38
Outcome:16 and 26 impaction Predictor: arch perimeter						
Intercept	6.4412	7.0312	…	…	…	…
X1	−0.0833	0.0891	−0.9349	0.3498	0.9201	0.77 a 1.10
Outcome: 16 and 26 impaction Predictor: arch length						
Intercept	7.8214	4.1347	…	…	…	…
X1	−0.2874	0.1490	−1.9283	0.0538	0.7502	0.56 a 1.00
Outcome:16 and 26 impaction Predictor: maxillary intermolar width						
Intercept	12.4353	5.9836	…	…	…	…
X1	−0.2902	0.1388	−2.0908	0.0365*	0.7481	0.57 a 0.98
Outcome: 16 and 26 impaction Predictor: maxillary intercanine width						
Intercept	5.4059	5.0581	…	…	…	…
X1	−0.1640	0.1511	−1.0853	0.2778	0.8487	0.63 a 1.14

* Statistically significant at *p* < 0.05

## Discussion

Based on the results of paired t-test and the low values obtained from the Dahlberg formula, it can be inferred that the method adopted for data collection was highly accurate and reliable, and the reproducibility analysis confirmed that the methodology used in this study demonstrated high consistency. The compatibility between the experimental and control groups regarding age, gender, mixed dentition stage, vertical malocclusions, and anterior mandibular crowding strengthens the validity of the study and reduces potential selection bias (Table 1).

*Unlike most previous studies that have focused mainly on radiographic and cephalometric analyses* [2, 4, 5], *the present investigation evaluated arch morphometric parameters using digital dental models. This approach allowed a more precise assessment of longitudinal and transverse arch dimensions*,* revealing significantly narrower arches in children with ectopic eruption.* The prevalence of ectopic eruption found in this study is consistent with literature, which reports rates between 0.75% and 4.3% [4, 5, 8]. Furthermore, ectopic eruption of first permanent molars was more frequent in the maxilla (84.37%) than in the mandible (15.63%), corroborating previous reports of maxillary predominance [4, 7]. Duncan and Ashrafi [1] found 89.7% of cases in the maxilla. With respect to unilateral or bilateral occurrence, there is no consensus in the literature. Sweet [23] reported a higher frequency of bilateral occurrence of ectopic eruption of maxillary first permanent molars, findings that were later confirmed by Martín-Vacas et al. [24], who found bilateral occurrence in 8.7% of cases. Conversely, O’Meara [25] reported conflicting results, finding no association between increased frequency and bilateral occurrence.

Children with ectopic eruption exhibited significantly reduced arch dimensions in both the maxillary and mandibular arches compared to controls (Table 2), particularly in intermolar and intercanine distances. These findings extend beyond the expected maxillary involvement, suggesting interdependent maxillomandibular effects during growth. These findings support the hypothesis proposed by Salbach et al. [26], who suggested that underdevelopment of the maxilla may contribute to eruption disturbances of the maxillary first permanent molars. Given the interdependence of maxillary and mandibular growth, maxillary deficiency may also explain the smaller measurements observed in mandibular arches. Maxillary hypoplasia can influence mandibular development through altered occlusal forces, functional adaptations, or compensatory mechanisms during the mixed dentition phase, leading to secondary arch constriction and increased crowding in the lower arch. This is consistent with prior reports [21, 26] linking eruption disturbances of first permanent molars to broader malocclusions, including mandibular crowding, lateral crossbites, and even tendencies toward mandibular prognathism due to adaptive growth patterns. These findings highlight the possibility of shared etiological factors or cascading effects on mandibular morphogenesis. Further longitudinal studies are warranted to elucidate these interarch relationships and their implications for early interceptive therapy.

Eruption disturbances in the first molar region may serve as an early indicator of dentitions at high risk for developmental discrepancies, potentially resulting in sagittal and transverse space deficiencies and even leading to Class III malocclusion [2, 4, 5]. In the present study, *maxillary intermolar width was identified as a significant predictor*,* highlighting a potential morphological predisposition.* Yaseen et al. [27] suggested that ectopic eruption may reflect an underlying disturbance in growth and development. Rah, Lee, and Ra [28] found ectopic eruption of maxillary first permanent molars in 93 children, 57% of whom also exhibited skeletal Class III malocclusion, a statistically significant difference compared to the control group, supporting the link between maxillary underdevelopment and ectopic eruption. More recently, Zhang et al. [5] reported insufficient maxillary development based on SNA angle analysis, as well as a significantly higher proportion of skeletal Class III malocclusion among affected children.

In the present study, the anteroposterior relationship of the arches according to Angle’s classification was not assessed, as all subjects were in the early mixed dentition stage. At this stage, molar relationships should be evaluated by the terminal plane of the second primary molars, since the relationship of the permanent first molars is not yet established. Although the terminal plane of the second primary molars serves as the primary reference for molar relationships at this stage, it was not systematically assessed due to ongoing exfoliation of the second primary molars in many participants, which removes the distal reference point and introduces measurement error.

No significant differences in molar angulation were found between groups, including subgroup analyses restricted to affected teeth (Table 3). Ectopically erupting molars showed approximately 5 degrees less distal angulation (increased mesial inclination). However, since these comparisons included all individuals in the experimental group — whether presenting ectopic eruption in maxillary right, left, or even in mandibular molars — it was necessary to conduct a more refined analysis. For this purpose, subgroups of only affected molars were analyzed. While this difference was not statistically significant, it represents a potential clinical trend that aligns with prior reports associating mesial angulation alterations with ectopic eruption risk [29–31]. In ectopic eruption, this distal angulation tends to be reduced, which may contribute to the condition. The lack of statistical significance reflect the limited number of events and inherent variability in angular measurements on panoramic radiographs, rather than a true absence of effect. Larger studies are needed to determine whether this trend holds clinical relevance.

A previous study [32] reported that in approximately 4% of children, the maxillary first molar deviates mesially during eruption, causing partial resorption of the roots of the second primary molar—characterizing ectopic eruption. Mendonça, Cuoghi, and Linhares [33] also found significantly reduced mesial angulation in ectopic cases, suggesting lack of space for proper eruption. Zhang et al. [5] reported a statistically significant difference of 4.16 degrees in molar angulations between ectopic and control groups, further confirming the clinical importance of mesial angulation in ectopic eruption.

Logistic regression analysis in the present study revealed a slight increase in the risk of impaction with each additional degree of mesial angulation of the maxillary right and left first molars. However, these associations were not statistically significant (*p* = 0.0919 and *p* = 0.1373, respectively) (Table 4). This may reflect the multifactorial nature of eruption trajectory. Literature likewise lacks consensus on the impact of angulation, due in part to differences in sample selection, age ranges, diagnostic methods, and clinical definitions across studies. Nevertheless, some authors have suggested altered eruption angulation as a potential etiological factor [33, 34]. Zhang et al. [5] also found significant associations between angulation and ectopic eruption using logistic regression.

No significant association was observed between the mesiodistal crown width of the primary second molars and the ectopic eruption of permanent molars maxillary right and left first molars (*p* = 0.1229 and *p* = 0.2177, respectively) (Table 4). This suggests that primary molar crown size does not influence adjacent permanent tooth eruption. Similarly, Zhang et al. [5] found no significant differences in crown width of maxillary primary second molars between groups. Although some studies proposed that larger crown dimensions of the permanent first molars or primary second molars may contribute to ectopic eruption [35, 36], further robust studies are required to clarify these potential associations.

Among arch perimeter, arch length, and transverse variables, logistic regression (Table 4) revealed that only upper intermolar width was significantly associated with ectopic eruption of maxillary first permanent molars. However, this finding is limited to the univariate analysis and should be interpreted as preliminary, given the high correlations among these arch measurements. These findings suggest that narrower arches may predispose to ectopic eruption by limiting eruption space and favoring mesial displacement and impaction of the permanent molars. *The higher prevalence of transverse malocclusion in the experimental group is consistent with the reduced maxillary arch dimensions observed and suggests that transverse deficiencies may represent a local contributing factor to ectopic eruption.* This result corroborates Salbach et al. [26], who linked maxillary underdevelopment to ectopic eruption of maxillary first molars and Pulver [34] that identified reduced maxillary dimensions as an etiological factor.

### Clinical implications

According to Chen et al. [5], ectopically erupting permanent first molars may remain asymptomatic and unnoticed by clinicians. Early detection of this anomaly can be achieved during routine radiographic examinations performed by the general dentist, pediatric dentist, or orthodontist, prior to the eruption of the maxillary permanent first molar, typically between 5 and 7 years of age. Clinicians should suspect ectopic eruption if a clinical delay exceeding six months is observed, or if there is pronounced mesial angulation of the erupting molar associated with clear signs of early resorption of the adjacent primary molars [17]. The findings of this study provide clinicians with evidence that children with underdeveloped maxilla should be carefully evaluated periodically, as they are more likely to develop ectopic eruption of first permanent molars. The observed reduction in arch dimensions, particularly maxillary intermolar width, suggests a morphological association with ectopic eruption, reinforcing the need for early assessment of arch measurements in children aged 5–9 years, without directly implying unmeasured clinical outcomes.

Early recognition of occlusal and anatomical features related to ectopic eruption enables timely orthodontic interception, thereby preventing adverse consequences such as severe root resorption of the second primary molar, premature exfoliation, and subsequent loss of arch space [13].

### Limitations and suggestions for future research

The use of panoramic radiographs, while clinically practical, is subject to image distortion, which may affect the precision of angular measurements. Moreover, because the data were drawn from orthodontic records of children seeking orthodontic treatment, the frequency recorded here may not fully reflect that of the general population. As records are cross-sectional, we cannot exclude prior self-corrected ectopic eruption in controls, a known phenomenon with rates ranging from 47% to 78% before age 7 [16]. Absolute measurements of arch morphology and tooth size were used to enable direct comparison with prior studies on ectopic eruption [2, 5, 26], which predominantly report linear values rather than proportional indices. The cross-sectional design precludes assessment of spontaneous correction rates or long-term outcomes, underscoring the need for longitudinal studies. Finally, *the retrospective nature of the study and the higher frequency of transverse malocclusion in the experimental group constitute potential confounding factors*,* which may influence the internal validity of the associations found*,* particularly regarding arch dimensions.*

## Conclusion

This study observed a frequency of 2.66% ectopic eruption of permanent first molars in a Brazilian orthodontic sample, with clear predominance in the maxilla. Individuals with the condition exhibited significantly reduced arch length and transverse dimensions in both arches. Maxillary intermolar width was the sole significant univariate predictor. These findings provide clinicians with evidence that narrower transverse arch dimensions may serve as an early indicator of ectopic eruption risk, supporting targeted radiographic screening and interceptive measures in the early mixed dentition.

## Data Availability

The data supporting the findings of this study were obtained from existing archival orthodontic records and are not publicly available due to ethical and privacy restrictions protecting patient confidentiality, in accordance with institutional ethics approval and applicable data protection regulations.
